# Effectiveness of a medical exercise prescription to promote physical activity in children: a pragmatic randomized trial in primary care

**DOI:** 10.3389/fped.2026.1732438

**Published:** 2026-02-10

**Authors:** Ángel Morillas-Mingorance, Ana Garach Gómez, Iván Gutiérrez García, Isabel Monereo Moreno, Francisco Javier Sánchez Ruiz-Cabello, Jose Maldonado

**Affiliations:** 1 Department of Pediatrics, University of Granada, Granada, Spain; 2 Centro de Salud Zaidín Sur, Distrito Sanitario Granada Metropolitano, Granada, Spain; 3 Department of Pediatrics, Hospital Universitario Virgen de las Nieves, Granada, Spain

**Keywords:** children, exercise prescription, physical activity, primary care, screen time

## Abstract

**Objectives:**

Most children fail to meet international physical activity (PA) recommendations. Can a single pediatric visit help reverse this trend? Brief counseling is infrequently used in clinical practice and its actual impact on children's activity levels remains uncertain. This study evaluates the impact of medical exercise prescriptions on children's PA levels compared to standard health advice. It also explores whether these prescriptions contribute to reducing daily screen time.

**Study design:**

A randomized controlled trial was conducted with 130 children aged 6–14 years. Participants were randomized into two groups: one received brief standard health advice (HA, 2 min), and the other received a 10-minute intervention including a written medical exercise prescription (MEP). All sessions were delivered by three trained pediatricians following a standardized protocol. PA levels (duration and intensity), screen time, and anthropometric data were collected through the same questionnaires. Physical activity was assessed at 3 and 12 months by the same investigator. Multivariate analyses were performed to evaluate changes over time.

**Results:**

Both interventions increased the duration and intensity of physical activity, with a significant increase in the number of children classified as physically active. An inverse relationship was observed between physical activity and screen time, indicating that promoting exercise may help reduce sedentary behavior. Parental satisfaction was high and similar in both groups, suggesting good acceptance of the interventions regardless of their format or duration.

**Conclusions:**

A short, focused message from a pediatrician—delivered in just a few minutes—can lead to lasting improvements in children's activity levels and screen habits. Brief health advice was as effective as personalized prescriptions, offering a simple, feasible and low-cost strategy to promote healthier lifestyles in primary care.

**Clinical Trial Registration:**

https://register.clinicaltrials.gov/prs/beta/studies/S000F96J00000036/recordSummary, identifier NCT06765460.

## Introduction

Physical activity (PA) during childhood is a key factor in long-term health. It contributes to the prevention of non-communicable diseases, increases muscle and bone strength, supports mental well-being, and improves overall quality of life (1–5).

The World Health Organization (WHO) and other prevention bodies highlight the benefits of health advice (HA) in promoting PA among patients, with exercise prescription as a relevant strategy to address sedentary behavior. They also recommend limiting screen use in children because of its association with physical inactivity and childhood obesity (6–9).

Sedentary lifestyles are linked to a wide range of health problems, including higher mortality, cardiovascular disease, obesity, hypertension, osteoporosis, and some cancers. Establishing global, evidence-based recommendations on physical activity for children and adolescents—adapted to individual capacities—is essential (4, 6–12). However, few studies have demonstrated, with sufficient methodological quality, the benefits of interventions aimed at increasing physical activity and reducing screen time in this population (13, 14).

In 2020, the World Health Organization (WHO) called for the integration of physical activity promotion and healthy diet counseling into routine primary care. In its guidelines, it recommends that children and adolescents aged 5–17 engage in at least 60 min of moderate- to high-intensity physical activity each day while limiting recreational screen time (8, 9). However, despite these clear recommendations, adherence remains low. Most children and adolescents still fail to meet these targets, showing low levels of physical activity and excessive screen exposure from the earliest stages of life (15).

These behaviors tend to cluster early, shaping lifestyle patterns that often persist over time (9, 16). Importantly, such early patterns are strongly influenced by the child's immediate social environment, particularly parental modeling, family routines, and opportunities for activity at home, school, and in the neighborhood (9, 16). Supporting this view, recent evidence from Spanish preschoolers shows that exceeding screen-time recommendations is associated with poorer health-related quality of life, whereas meeting physical-activity guidelines improves well-being (4).

The Spanish Association of Pediatrics (AEP) echoes this guidance, advising children in this age group to include both aerobic activity and muscle- and bone-strengthening exercises at least three times per week (17). Other national bodies, such as Previnfad, recommend introducing these habits during standard health check-ups from the age of two (10, 18). Similarly, the AEP suggests limiting daily screen use in children to under two hours and replacing sedentary time with active play or other physical routines (7, 10, 17–21).

International experiences have shown that exercise prescriptions can be effectively implemented in primary care. New Zealand's Green Prescription program—one of the longest-running national initiatives—enables healthcare providers to prescribe physical activity with individualized follow-up. Long-term evaluations have reported sustained increases in activity levels, better mental well-being, and improved quality of life up to three years after participation (22–25). These results highlight the potential of structured, follow-up–based prescriptions to promote lasting behavioral change in adults.

Recent meta-analytic evidence confirms that health professional–led interventions effectively increase physical activity in adults, reinforcing the rationale for adapting this approach to pediatric care (26).

Healthcare professionals play a central role in helping families adopt active lifestyles, particularly by engaging parents and supporting changes in daily routines. To promote PA among children, two strategies are most frequently used in clinical settings: brief health advice (HA) and the medical exercise prescription (MEP). These approaches differ in their level of personalization, duration, intensity, and the extent of follow-up involved (12).

HA involves brief counseling based on international recommendations, aimed at motivating patients to increase PA in their daily routines. It is typically delivered within a few minutes during a standard medical visit. In contrast, MEP involves additional time to create a tailored plan that considers the child's physical condition, motivation, and social context. It also includes setting specific goals and planning structured follow-up. Ideally, MEP should be provided by a trained healthcare professional (12).

Evidence on the effectiveness of primary care interventions to promote PA in children and adolescents is still limited (5, 12, 19, 27–30). Although numerous school, family, and community programs have sought to increase children's activity levels, their effects have generally been modest and short-lived (28–30). Few studies have tested strategies that can be realistically applied in children. Therefore, this trial was designed to address that gap by comparing two feasible approaches to promote PA and reduce screen time during routine pediatric consultations.

We hypothesized that both strategies would improve PA and reduce screen time over time, and that MEP would not differ significantly from HA in this pragmatic setting.

## Material and methods

### Design

**Figure 1:**
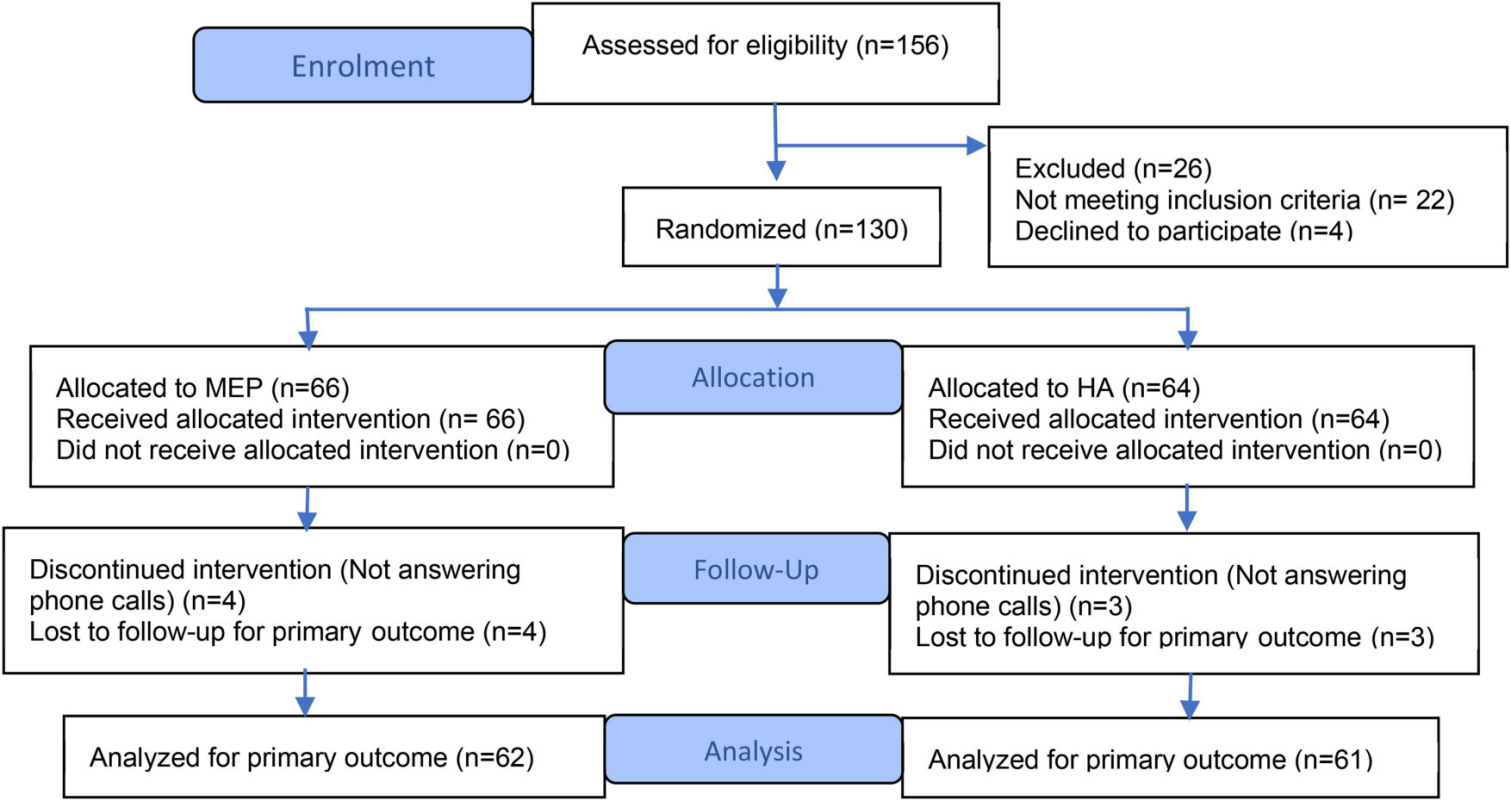
Algorithm for the distribution and participation of the study population. Adapted from the CONSORT 2025 statement (31).

This was a single-blind, parallel, two-group clinical trial conducted in pediatric primary care. Participants were recruited consecutively during routine pediatric primary care visits, as described below. Children were randomly assigned to receive either an individualized medical exercise prescription (MEP) or brief health advice (HA). Block randomization was used to ensure balanced group sizes (STATA version 16.1). This randomized controlled trial was conducted and reported in accordance with the CONSORT 2025 Statement. A flow diagram summarizing participant progress is shown in Figure 1 (31).

An independent investigator generated the randomization sequence and scheduled eligible patients for their appointments. Each assignment was placed in a sealed, opaque envelope, opened by the pediatrician immediately before the consultation, ensuring concealment of the allocation sequence until the moment of intervention.

Three pediatricians trained in both interventions conducted the sessions following a shared protocol. Families were unaware that two types of intervention existed. Follow-up evaluations at 3 and 12 months were carried out by the same blinded investigator to maintain consistency in data collection.

### Sample size, recruitment and eligibility criteria

Participants were recruited consecutively during routine pediatric primary care visits in two primary care groups of an urban health center in Spain. Children attending scheduled in-person consultations for any reason were assessed for eligibility by their pediatrician and invited to participate. No targeted outreach, advertising, or active recruitment strategies were used. Written informed consent was obtained from parents or legal guardians prior to enrollment.

Pediatric patients aged 6–14 years attending in-person consultations were eligible for inclusion. Exclusion criteria included chronic or complex medical conditions contraindicating moderate or vigorous physical activity. As summarized in Figure 1, 130 participants were randomized and allocated to the MEP (*n* = 66) or HA (*n* = 64) groups. All randomized participants received the allocated intervention, and the primary outcome analysis included 62 participants in the MEP group and 61 in the HA group.

### Study variables and patient classification

#### Effect variables

Primary outcomes included PA indicators: average daily PA time (in minutes), number of active days per week, intensity (light, moderate, or vigorous), PA classification (based on time and intensity), and ST (daily minutes by device: mobile phone, tablet, computer, or television).

#### Independent variables

Sociodemographic data included age, sex, nationality, address, number of siblings, and parental educational level (primary, secondary, or higher). Anthropometric variables were weight, height, body mass index (BMI) and waist circumference. Additional factors included academic performance (grade repetition or parental perception), parental PA (average daily minutes of father and mother), mobile phone ownership, and satisfaction with the intervention. Satisfaction and motivation to change PA and ST habits were rated on a 5-point Likert scale. Parents' perception of the need to prevent sedentary behavior in PC was also recorded.

#### Patient classification

Patient classification was based on physical activity data collected using an adapted version of the International Physical Activity Questionnaire for Adolescents (IPAQ-A) (32), administered at baseline and at 3- and 12-month follow-up assessments. The questionnaire was adapted for use across the study age range, with age-appropriate examples and parental assistance when needed to support comprehension and reporting accuracy.

Physical activity was assessed with reference to a typical, usual week. Information on frequency (days per week), duration (minutes per day), and intensity (light, moderate, or vigorous) was collected according to the type of reported activity.

PA levels were classified using an intensity-weighted approach: light activity (minutes weighted ×0.3), moderate activity (×0.6), and vigorous activity (×1.0). Intensity categories were defined using functional descriptors. Walking or activities performed while maintaining conversation were considered light. Moderate PA included activities such as dancing, jogging, swimming, or paddle tennis, in which conversation became difficult. Vigorous PA included football, basketball, or intense running, characterized by marked tachycardia, sweating, and fatigue.

Based on the weighted weekly minutes of PA, participants were initially categorized into four groups according to WHO criteria: active (>400 effective minutes/week), moderately active (251–400 min/week), partially active (60–250 min/week), and inactive (<60 min/week). For analytical purposes, categories were later dichotomized into active (>250 min/week) and inactive (<250 min/week).

### Intervention and follow-up

#### HA group (control)

Participants received brief counseling on the benefits of performing at least one hour of daily physical activity and limiting recreational screen time. The advice was delivered verbally by a pediatrician during the clinical visit. The session lasted less than two minutes and focused on general lifestyle recommendations, without individualized planning or structured follow-up. Families were also given an informational WHO factsheet summarizing age-specific physical activity targets and screen time recommendations.

#### MEP group (intervention)

Participants received a structured, individualized exercise prescription prepared and delivered by a trained pediatrician in motivational interviewing and behavioral counseling. The MEP was designed as a pragmatic intervention tailored to each child and family. Individualization was achieved by integrating the child's baseline activity level, preferences, and motivation with family-specific factors such as available time, financial resources, and access to physical activity opportunities. The emphasis was placed on identifying realistic and sustainable options that could be incorporated into daily routines within each family's context. The consultation lasted approximately 10 min and followed a standardized protocol to ensure consistency across participants. The intervention included seven steps:

Baseline classification and feedback. Each child's physical activity level was assessed and categorized as active, partially active, or inactive. This classification was shared with the family during the visit to increase awareness and engagement, serving as the starting point for goal setting. In the HA group, this assessment was recorded but not communicated.
Motivation and readiness assessment. The pediatrician briefly explored each participant's and family's motivation, perceived barriers, and willingness to modify habits using open-ended questions.
Activity preferences. Children were asked about their preferred types of exercise or sports to ensure engagement and feasibility.
Feasibility assessment. The pediatrician discussed opportunities for incorporating physical activity into the child's weekly schedule, considering time availability, intensity, access to safe environments and family resources.
Goal setting. Specific and realistic goals were proposed regarding type, frequency, duration, and intensity of activities, aligned with WHO standards.
Personalized exercise plan. Families received a written plan summarizing the agreed objectives and practical recommendations. The plan emphasized short-term achievable goals and included both structured activities (e.g., organized sports) and unstructured ones (e.g., active play, walking or cycling to school or home-based routines). When appropriate, families were also encouraged to use freely available online resources suitable for children.
Follow-up. Families were advised to monitor adherence, and the plan was reviewed in 3 months. Parental involvement was emphasized as a central element in modeling and maintaining active behaviors.

Those classified as active were encouraged to maintain their current activity level. For participants categorized as partially or moderately active, the goal was to reach active status by increasing PA by one to two additional days per week and incorporating daily routines involving movement. For inactive participants, PA was introduced progressively, with a structured plan aimed at achieving at least partial activity in the short term and active status as a long-term goal.

Follow-up assessments were conducted by telephone at 3 and 12 months. The same questionnaire was used in both groups. Satisfaction and motivation variables were assessed only at 3 months to minimize recall bias. Both interventions were delivered within standard pediatric consultations, maintaining the typical time and environment of routine care.

### Data collection and statistical analysis

Data were collected through medical records, physical examinations, and a structured questionnaire completed by the pediatrician. A password-protected database was created, accessible only to the investigators. Identifying information was stored in a separate, secure database available exclusively to the principal investigator. Data confidentiality was ensured in accordance with current data protection regulations.

Descriptive analyses were performed for all study variables. Measures of central tendency and dispersion were calculated for continuous variables, and absolute and relative frequencies for categorical variables. The Kolmogorov–Smirnov test was used to assess the normality of continuous variables.

To compare quantitative outcomes between groups, Student's *t*-test or Mann–Whitney U-test was applied as appropriate. Categorical variables were compared using the chi-squared test. Changes in PA time, ST, and number of PA days per week over time were analyzed using linear mixed-effects models. For PA intensity (none/low vs. moderate/vigorous) and PA classification (≤250 min vs. >250 min/week), logistic regression models were applied. Fixed-effect terms included time point (baseline, 3 and 12 months), group (HA or MEP), and their interaction.

The interaction between time and group was not statistically significant in any model; therefore, the group variable was excluded from the final models. Pairwise comparisons were performed across time points with Bonferroni correction. Additional models included sociodemographic and clinical covariates as fixed effects. Final multivariate models were constructed by including variables with *p* < 0.200 in bivariate analyses, retained or excluded using the likelihood ratio test, with *p* ≥ 0.05 as the exclusion threshold.

All analyses were conducted using STATA version 16.1. All figures and tables were generated using RStudio (R version 4.5.0).

## Ethical aspects

The study was approved by the Biomedical Research Ethics Committee on January 15, 2023, and conducted in accordance with the ethical principles of the Declaration of Helsinki and current data protection regulations. Written informed consent was obtained from parents or legal guardians, and assent was requested from children aged 12 years and older.

## Results

The bivariate analysis revealed no significant differences between MEP and HA groups across sociodemographic, anthropometric, lifestyle, or activity-related variables (Table 1). The randomization produced two well-balanced groups, reducing baseline confounding.

**Table 1:** Bivariate descriptive analysis of the population.

Variables	MEP (*n* = 66)	HA (*n* = 64)	*p* value
Qualitative variables *n* (%)			
Gender (Female)	26 (39%)	30 (47%)	0.389
Nationality (Spanish)	57 (86%)	50 (78%)	0.218
Owns mobile phone (Yes)	28 (42%)	29 (45%)	0.740
Level of education (Father)			
Primary school	25 (40%)	35 (55%)	0.219
High school/vocational training	23 (36%)	19 (30%)	
University	15 (24%)	10 (15%)	
Level of education (Mother)			
Primary school	20 (30%)	24 (38%)	0.125
High school/vocational training	32 (49%)	20 (32%)	
University	14 (21%)	20 (32%)	
Daily time spent by both parents on PA			
Do not do	39 (59%)	33 (52%)	0.615
Less than 1 h	13 (20%)	17 (27%)	
Between 1 and 2 h	14 (21%)	13 (20%)	
More than 2 h	0 (0%)	1 (1%)	
Quantitative variables			
Children age (years)			
Median (Interquartile Range)^b^	10 (5)	9 (4)	0.697
Mean ± sd	9.56 ± 2.34	9.40 ± 2.52	
BMI			
Median (Interquartile Range)^b^	18 (6)	18 (7)	0.537
Mean ± sd	19.98 ± 4.35	19.85 ± 5.04	
PA Days/Week Mean ± sd^a^	2.29 ± 1.48	2.22 ± 1.67	0.890
PA time/Week (minutes) Mean ± sd^b^	172.90 ± 165.96	151.42 ± 130.43	0.719
ST/day (minutes) Mean ± sd^b^	163.34 ± 83.72	152.51 ± 84.05	0.768

a T-student Test.

b U-Mann–Whitney Test.

Table 2 shows the fixed effect estimates for the comparison between MEP and HA groups, adjusted for time. No significant differences were observed for any of the outcomes assessed, including weekly PA time, number of PA days, PA intensity, PA classification, or daily screen time. The two groups followed comparable trajectories throughout the study period.

**Table 2:** Linear/logistic mixed model estimates for the fixed effect of group (MEP group vs. HA group), adjusting for the fixed effect of time.

Variables	*p* value	Coefficient	95% CI	
PA total time	0.880	−4.360	−60.85	52.13
PA Days/Week	0.760	−0.063	−0.47	0.34
ST total time	0.598	5.079	−13.78	23.94
	*p* value	OR	95% CI	
PA intensity (none-low, moderate-high)	0.568	0.764	0.30	1.93
PA classification (0–250 min, >250 min)	0.773	0.764	0.58	2.08

95% CI (95% Confidence Interval) OR (Odds Ratio).

**Figure 2:**
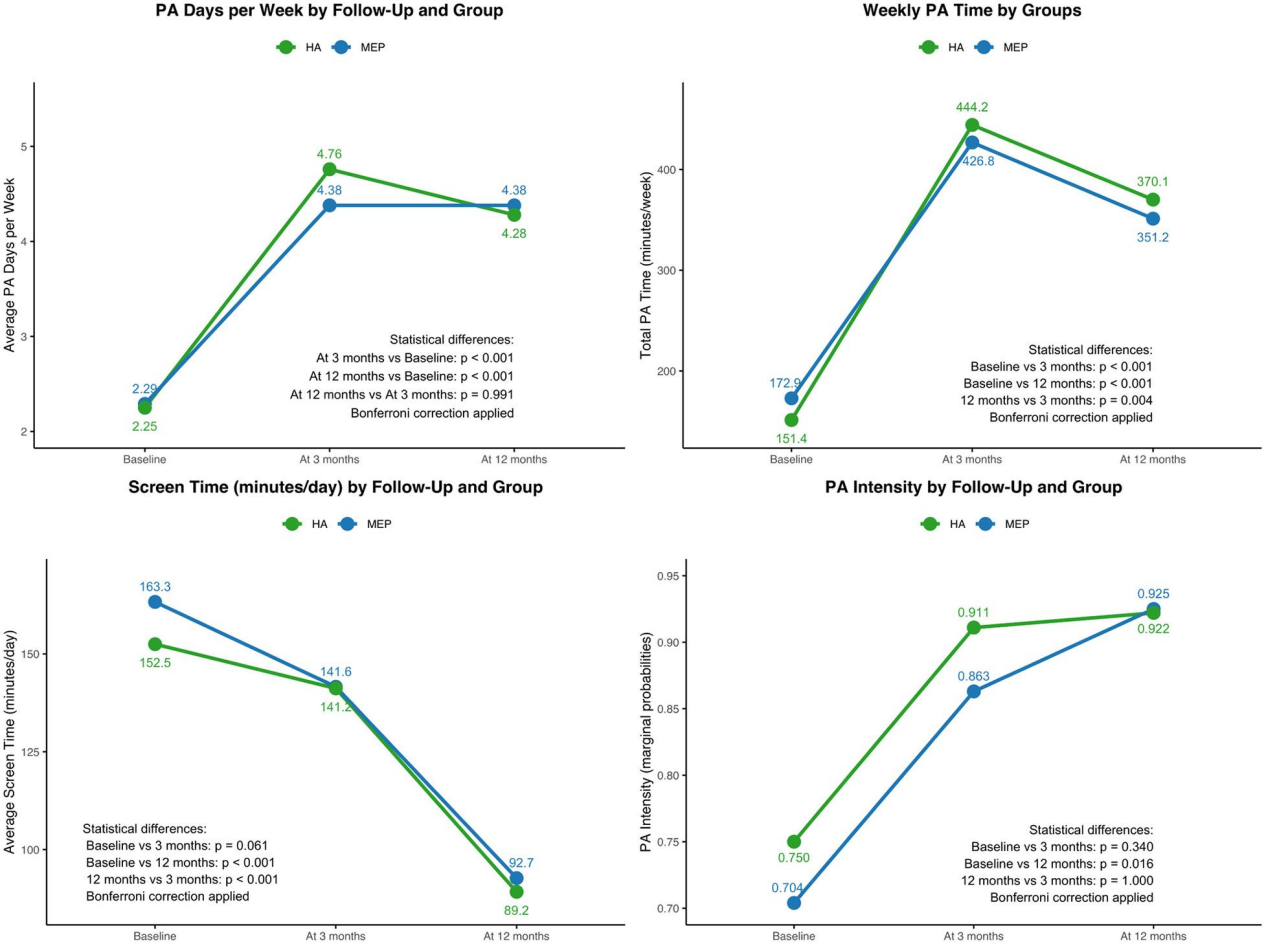
Time course evolution of PA time, PA days per week, ST and PA intensity: marginal predicted means and probabilities for the intervention and control groups.

Figure 2 shows the time-course evolution of the primary outcomes—PA time, PA days per week, PA intensity, and screen time—through marginal predicted means and probabilities for each group at baseline, 3 months, and 12 months.

Both MEP and HA groups showed significant increases in PA time and PA days per week at 3 and 12 months compared to baseline, with no significant difference between the two follow-up points. PA intensity also increased significantly over time in both groups. For screen time, a significant reduction was observed only between baseline and 12 months, while no relevant change occurred at 3 months. Bonferroni correction was applied to account for multiple comparisons.

### Physical activity time

Table 3 presents the multivariate model for total weekly PA time. Female sex was associated with a significant reduction [−70.6 min per week; 95% CI (−118.95, −22.20), *p* = 0.004]. Screen time showed a negative, though not statistically significant, association [−0.27 min per ST minute; 95% CI (−0.56, 0.02), *p* = 0.071]. In contrast, engaging in moderate-to-vigorous PA was strongly associated with greater weekly activity [+211.1 min; 95% CI (153.79, 268.45), *p* < 0.001].

**Table 3:** Multivariate analysis of factors influencing weekly PA time.

Variable	Coefficient (Coef)	*p*-value	95% Confidence Interval
Sex (Female)	−70.574	0.004	[−118.95, −22.20]
Screen Time	−0.269	0.071	[−0.56, 0.02]
Exercise Intensity (Mod-High)	211.118	0.000	[153.79, 268.45]
Follow-Up (at 3 months)	234.328	0.000	[189.73, 278.92]
Follow-Up (at 12 months)	137.933	0.000	[90.62, 185.25]

Compared to baseline, PA time increased significantly at both follow-up points: +234.3 min at 3 months [95% CI (189.73, 278.92), *p* < 0.001] and +137.9 min at 12 months [95% CI (90.62, 185.25), *p* < 0.001]. These results reflect the contribution of PA intensity and follow-up period to increases in weekly activity.

As shown in Table 3, the number of days per week dedicated to PA increased significantly in both groups compared to baseline. The average increase was 2.27 days at 3 months [95% CI (1.91, 2.63), *p* < 0.001] and 2.02 days at 12 months [95% CI (1.70, 2.42), *p* < 0.001].

In the bivariate analysis, BMI showed a significant inverse association with total PA time: for each unit increase in BMI, PA decreased by 6.48 min per week [95% CI (−12.85, −0.12), *p* = 0.046]. No significant differences in PA levels were observed by age (*p* = 0.086), parental education (father: *p* = 0.982; mother: *p* = 0.230), or mobile phone ownership (*p* = 0.173).

### Intensity of physical activity

As shown in Figure 2, PA intensity increased significantly in both groups at 3 and 12 months compared to baseline (*p* < 0.001). In the multivariate analysis (Table 4), total PA time was positively associated with moderate-to-vigorous intensity [OR = 1.03 per minute; 95% CI (1.02, 1.04), *p* < 0.001], and higher paternal education level was also a significant predictor [OR = 3.91; 95% CI (1.93, 7.93), *p* < 0.001].

**Table 4:** Multivariate analysis of factors influencing PA intensity.

Variable	Odds Ratio	*p*-value	95% Confidence Interval
Father's Education Level (High)	3.91	0.00	[1.93, 7.93]
Total PA Time (Minutes)	1.03	0.00	[1.02, 1.04]

In the bivariate analysis, maternal educational level was significantly associated with PA intensity [OR = 1.95; 95% CI (1.15, 3.33), *p* = 0.014], but this association did not persist in the multivariate analysis. PA intensity was inversely related to screen time [OR = 0.99; 95% CI (0.98, 0.99), *p* < 0.001], and children without mobile phones were more likely to engage in moderate-to-vigorous PA [OR = 2.48; 95% CI (1.14, 5.37), *p* = 0.021]. Lower BMI was also associated with higher PA intensity in the bivariate analysis [OR = 0.91; 95% CI (0.84, 0.98), *p* = 0.020], but this association was not independent after adjustment. No significant relationship was found for sex [OR = 2.09; 95% CI (0.97, 4.52), *p* = 0.060].

### Physical activity classification

The multivariate analysis (Table 5) showed that male sex was significantly associated with achieving the active PA classification (>250 min/week) [OR = 3.14; 95% CI (1.69, 5.82), *p* < 0.001]. Screen time was inversely associated [OR = 0.99 per minute; 95% CI (0.99, 1.00), *p* = 0.015].

**Table 5:** Multivariate analysis of factors influencing PA classification.

Variable	Odds Ratio	*p*-value	95% Confidence Interval
Sex (male)	3.14	0.00	[1.69, 5.82]
Screen Time (minutes)	1.00	0.01	[0.99, 1.00]
Follow-Up (at 3 months)	11.54	0.00	[5.60, 23.79]
Follow-Up (at 12 months)	8.06	0.00	[3.89, 16.71]

Compared to baseline, the probability of being classified as active increased substantially at follow-up: OR = 11.54 at 3 months [95% CI (5.60, 23.79), *p* < 0.001] and OR = 8.06 at 12 months [95% CI (3.89, 16.71), *p* < 0.001].

The proportion of children classified as active (>250 min of PA per week) increased substantially over time. At 3 months, 70.6% of participants met this threshold, compared to 25.2% at baseline (*p* < 0.001). A similar improvement was observed at 12 months, with 68.5% classified as active vs. 23.9% at baseline (*p* < 0.001). These changes reflect a consistent and significant shift in PA classification during follow-up.

### Screen time

The multivariate analysis (Table 6) showed that older age was significantly associated with higher daily screen time [Coef. = 5.12 min per year; 95% CI (1.36, 8.88), *p* = 0.008]. In contrast, engaging in moderate-to-high PA intensity was associated with a reduction in screen exposure [Coef. = −26.24 min per day; 95% CI (−45.30, −7.17), *p* = 0.007]. Screen time also decreased significantly at 12 months compared to baseline [Coef. = −61.76; 95% CI (−75.81, −47.72), *p* < 0.001], while the reduction at 3 months did not reach statistical significance [Coef. = −12.02; 95% CI (−26.27, 2.22), *p* = 0.098].

**Table 6:** Multivariate analysis of factors influencing screen time.

Variable	Coefficient (Coef)	*p*-value	95% Confidence Interval
Age	5.12	0.01	[1.36, 8.88]
PA Intensity (Mod-High)	−26.24	0.01	[−45.30, −7.17]
Follow-Up (at 3 months)	−12.02	0.10	[−26.27, 2.22]
Follow-Up (at 12 months)	−61.76	0.00	[−75.81, −47.72]

In the bivariate analysis, mobile phone ownership was associated with higher screen time [Coef. = 26.09; 95% CI (7.11, 45.06), *p* = 0.007]. Both total PA duration and intensity showed inverse correlations with screen time, and lower screen exposure was also associated with a higher PA classification [OR = 0.99; 95% CI (0.98, 0.99), *p* < 0.001]. No significant differences in screen time were observed by sex (*p* = 0.517) or parental education level (mother: *p* = 0.116; father: *p* = 0.085).

### Satisfaction

Satisfaction with the intervention was high in both groups. At 3 months, 83.3% of participants in the MEP group and 76.8% in the HA group considered the counselling useful, with no statistically significant difference (*p* = 0.377). A weak but positive correlation was observed between satisfaction and weekly PA time (Spearman's *ρ* = 0.29), suggesting that more satisfied children tended to be more active.

## Discussion

This trial confirms that both HA and MEP effectively increased weekly physical activity in children. Beyond the rise in duration and frequency, more participants achieved the active classification threshold over time. We also found an inverse association between physical activity and screen time: children who spent more time in front of screens tended to be less active. This pattern, confirmed in multivariate analysis, suggests that reducing screen time may help promote healthier routines. Other studies have described this link, but when interventions took place in schools or homes, their impact was often modest (27, 28, 33–36). Most available evidence on school and community-based interventions—whether from trials or reviews—has shown some effect, but overall impact remains limited (30, 37–40).

In primary care, most studies have been rated as moderate to low quality in systematic reviews (26, 28, 29). Still, our findings align with prospective trials showing that exercise prescriptions can increase physical activity in children (5, 14). Compared to Ford's four-week follow-up, our 12-month design adds value in terms of long-term outcomes (14). Ortega-Sanchez et al. also reported 12-month data, but their multicenter approach and focus on older adolescents introduced variability (5). Our single-center design, with all sessions conducted by three trained pediatricians under a standardized protocol, ensured procedural consistency and strengthened internal validity.

Satisfaction levels were similar in both groups, suggesting that families responded positively regardless of the format or duration of the recommendation. This acceptance reinforces the practical value of integrating physical activity advice into routine pediatric care.

These findings support the implementation of physical activity advice in routine primary care. Both HA and MEP increased PA levels and sustained these improvements over time. Given this consistency, setting minimum standards for their delivery seems both feasible and necessary.

In practice, limited consultation time and doubts about the effectiveness of current recommendations often lead to inconsistent use. Offering clear, structured guidance may help overcome this barrier and integrate PA promotion into daily clinical work (25).

A short message delivered in just 2 min was enough to improve physical activity and reduce screen time over time. This effect likely reflects the trust many families place in their pediatrician or family doctor—a figure often seen as close, credible, and worth listening to. This effect may be mediated by family engagement, including parental support, shared routines at home, and the feasibility of incorporating physical activity into the child's daily environment. In primary care, this relationship may be key to sustained behavior change and long-term adherence.

MEP also maintained physical activity improvements over time. However, as it requires more time and involvement than HA, its use may be less efficient in routine consultations. Even so, its potential remains valuable, especially for children who require closer follow-up or additional motivation to maintain healthy habits. Delivered twice a year, MEP could reinforce recommendations, offer structure to unresponsive patients, and support those needing continued guidance. Exploring this approach through new trials would help define its role within broader primary care strategies against sedentary lifestyles and pediatric obesity.

Physical activity during school hours was not included in the analysis because of the wide variability among schools and programs. In some cases, physical education lessons focused mainly on theory or low intensity tasks, which could make comparisons between participants less reliable.

Another methodological consideration relates to the assessment of PA. Reported activities were not converted into metabolic equivalent tasks (METs), as the primary aim of the study was to evaluate changes in physical activity patterns over time rather than to estimate precise energy expenditure. This approach is consistent with the pragmatic design of the trial and its implementation in routine pediatric primary care.

Although more comprehensive, child-specific questionnaires are available and may provide a more detailed characterization of PA behaviors, their length and complexity can limit feasibility in busy clinical settings. We therefore used a brief adapted version of the IPAQ-A, which allowed repeated assessment over time with minimal burden in routine pediatric primary care.

Beyond this methodological aspect, several contextual limitations should also be acknowledged. Families who attend routine pediatric visits may differ from those who do not, which could influence the generalizability of the findings. Families living in disadvantaged areas often face barriers to accessing healthcare, transportation, or safe spaces for physical activity (25). Similar trends have been described in other countries, where families with fewer resources participate less in preventive and health promotion programs (40, 41). Yet these children may also benefit from brief counseling or an exercise prescription. Strengthening collaboration between healthcare teams, schools, and community organizations could help reduce these gaps and extend the benefits of brief interventions to more vulnerable families (42).

A key limitation of this study lies in its single-center design. Nonetheless, the intervention is easily replicable, and future multicenter trials could enhance heterogeneity and improve external validity. This study may serve as a starting point for developing standardized protocols and national guidelines for physical activity counseling and prescription in pediatric primary care.

Another limitation involves follow-up data collection, which relied on telephone interviews at 3 and 12 months. This method may introduce recall bias. In addition, self-reported data from children and parents could have introduced social desirability bias, possibly leading to an overestimation of physical activity or underestimation of screen time. However, using the same blinded investigator throughout follow-up helped minimize this effect and maintain internal validity.

Objective measures of physical activity, such as accelerometers or pedometers, were not used, which could have provided more precise estimates of activity levels. Additionally, objectifiable variables such as blood pressure or daily steps taken could have been measured, although this would have required electronic devices and much more stringent monitoring. Nevertheless, current evidence on the use of technology to monitor PA in children and adolescents remains insufficient (43–45).

This trial is among the few randomized controlled studies on physical activity and screen time in pediatric primary care. Its design captured both short- and long-term outcomes: improvements observed at 3 months persisted at 12 months. Contrary to initial expectations, PA levels did not decline over time, but remained above baseline. Our findings support the call by Sallis et al. for integrating evidence-based strategies into clinical and community settings to close the gap between knowledge and practice (46).

The sample (*n* = 130) included children with varied anthropometric and sociodemographic profiles, reflecting the diversity of the general pediatric population and supporting the external validity of our findings. Moreover, classifying physical activity by time and intensity (>250 vs. <250 effective minutes per week) allowed us to capture meaningful changes in activity levels throughout the study.

Our findings suggest that even brief counseling by a pediatrician—lasting just 2 min—can lead to sustained improvements in physical activity and screen time. In our context, standard health advice was as effective as a medical exercise prescription and represents a practical, low-cost approach to support healthier routines in children during routine primary care visits.

These results underscore the role of primary care professionals in shaping pediatric health through concise, well-delivered interventions. Taken together, these findings highlight the opportunity for pediatricians to integrate structured physical activity counseling into everyday consultations as a practical tool for sustained behavior change and health promotion.

Promoting healthier habits in pediatric care does not require complex strategies: a few minutes of clear, structured counseling during routine visits can foster lasting behavior change and help reduce sedentary time; it happens right where it matters most—the pediatric visit, a unique opportunity to offer trusted, evidence-based advice and support healthier routines in children. Future research should explore how to extend these benefits to other primary care settings and adapt interventions for children with greater needs.

## Data Availability

The original contributions presented in the study are included in the article/Supplementary Material, further inquiries can be directed to the corresponding author.
